# The Radcliffe Infirmary, Oxford

**Published:** 1891-05-16

**Authors:** 


### EDITOR'S LETTER-BOX.

[Our correspondents are reminded that prolixity is a great bar to publication, and that brevity of style and conciseness of statement greatly facilitate early insertion].

### THE RADCLIFFE INFIRMARY, OXFORD.

Miss M. Hayter writes from Rome with regard to the Radcliffe Infirmary, Oxford, as follows: "I must add my humble protest to the letter sent you by the physicians and surgeons of the Radcliffe Infirmary, Oxford. I was a resident exactly opposite the building for six years, and for some time a daily worker there. I therefore have reason to know how misleading the statements lately made in The Hospital have been. Everybody in Oxford knows of the self-denying efforts of Dr. Bright, the busy head of a college, on behalf of the institution, and of the great interest taken by all classes of people in Oxford in the welfare and amusement of the patients. Until lately, when I have been absent from Oxford, I understood that the children had plenty of visitors also. As to antiseptics, surely that is the province not of the treasurer, but of the surgeons. Lister's method has been efficiently carried out in the wards by Mr. Horatio Symonds. As to the spray lately so minutely described in the pages of The Hospital, I have been told that it is quite obsolete now in the London hospitals, and never used by Lister himself. I quite understand that your Commissioner may have found plenty to criticise unfavourably in an old building, but without a lengthened residence in Oxford he could form no impartial judgment of the working of the Radcliffe Infirmary.

